# Identifying genetic predictors of depression risk: 5-HTTLPR and *BDNF* Val66Met polymorphisms are associated with rumination and co-rumination in adolescents

**DOI:** 10.3389/fgene.2013.00246

**Published:** 2013-11-13

**Authors:** Lindsey B. Stone, John E. McGeary, Rohan H. C. Palmer, Brandon E. Gibb

**Affiliations:** ^1^Department of Psychiatry, Brown University Alpert Medical SchoolProvidence, RI, USA; ^2^Department of Psychology, Binghamton UniversityBinghamton, NY, USA; ^3^Providence VA Medical CenterProvidence, RI, USA; ^4^Division of Behavioral Genetics, Rhode Island HospitalProvidence, RI, USA

**Keywords:** depression, rumination, BDNF Val66Met, 5-HTTLPR, co-rumination

## Abstract

**Background:** Despite research supporting moderate heritability of depression, efforts to replicate candidate gene associations to depression have yielded inconsistent results. We tested whether Val66Met and 5-HTTLPR exhibit utility as genetic markers of depression risk, testing for replicable associations to cognitive and interpersonal endophenotypes of depression (rumination and co-rumination), and further exploring developmental and sex moderation.

**Method:** In Study I, 228 youth (ages 8–14) of mothers with or without a history of MDD during the child's lifetime were recruited from the community. Replication tests were carried out in Study II, a sample of 87 youth with similar recruitment.

**Results:** In Study I, the Val66Met single-nucleotide polymorphism (SNP) was associated with rumination in adolescents, but not children, such that adolescents homozygous for the Val allele reported higher rumination levels. Further, a cumulative genetic score (CGS) (Val66Val and 5-HTTLPR) predicted higher levels of co-rumination, specifically among adolescent girls. Both genetic associations maintained significance after covarying for current depressive symptomology, and the other endophenotype. Finally, both genetic associations exhibited similar effect sizes in Study II, although results did not reach statistical significance.

**Conclusions:** Results replicate a previously reported association between the brain derived neurotrophic factor (*BDNF*) Val allele and rumination in adolescents, and provide preliminary support for a CGS predictive of co-rumination in adolescent girls. The current study indicates that candidate genes may demonstrate utility as consistent genetic markers of depression risk when focused on specific phenotypes, and supports the need to explore potential differential effects of developmental stage and sex. However, given the small sample sizes and possibility of chance findings, these results should be interpreted with caution pending replication.

## Introduction

Major depressive disorder (MDD) is one of the more common mental illnesses, with a lifetime prevalence rate of 16.2% (Kessler et al., 2003), and is the leading cause of disability worldwide (WHO, 2012). The complex presentation of MDD, with somatic, cognitive, and affective impairments makes it likely that many genes are involved in transmission and vulnerability (Nestler et al., 2002; Demirkan et al., 2010; Lubke et al., 2012). Although depression rates markedly increase across adolescence and young adulthood (Avenevoli et al., 2008), genetic studies in youth are lacking. The current study tests whether the brain derived neurotrophic factor (*BDNF*) Val66Met variant and 5-HTTLPR, *two* of the most widely studied candidate polymorphisms, exhibit replicable associations to established cognitive and interpersonal endophenotypes of depression.

In the search for genetic markers of depression risk, it is likely that prior inconsistencies are partially attributable to the selection of psychiatric phenotypes, which are often heterogeneous in diagnostic presentation. One alternative to diagnoses is to focus instead on endophenotypes, which are less complex traits closer to the mechanism of gene action, and thus may more successfully identify the genetic underpinnings of psychiatric disorders (Gottesman and Gould, 2003). In the context of depression, results from a recent twin study with adolescents supports that one cognitive phenotype, the tendency to ruminate, is partially heritable (21%; Moore et al., 2013). Rumination reflects the tendency to perseverate on one's negative mood, symptoms, and the potential causes and consequences of distress (Nolen-Hoeksema, 1991). There is considerable evidence that rumination predicts the development and maintenance of depression in both youth and adults (for review, see Nolen-Hoeksema et al., 2008). Importantly, Moore and colleagues' twin study found that heritable influence largely accounted for the association between rumination and depression. Taken together, these results suggest that rumination is an established, heritable risk factor, making it an ideal phenotype for testing genetic associations for depression risk.

The selection of a genetic predictor of rumination was driven by a recent finding indicating that *BDNF* is associated to rumination in adolescents (Hilt et al., 2007). *BDNF* is a protective protein responsible for neuronal growth, development, and synaptic plasticity. Lower expression of *BDNF* has been implicated in depression vulnerability such that the antidepressant effect seen with psychotrophic treatments is attributed to increasing *BDNF* levels, which theoretically restores hippocampal functioning (for review see Nestler et al., 2002). An amino acid substitution (valine to methionine) at codon 66 (Val66Met) is the most widely studied *BDNF* single-nucleotide polymorphism (SNP) in genetic association studies with depression. Support for an association between *BDNF* and depression risk in adults has been inconsistent (e.g., Sklar et al., 2002; Jiang et al., 2005 vs. Gratacos et al., 2007; Chen et al., 2008). In contrast, studies examining mood and anxiety disorders in youth including: obsessive compulsive disorder (Hall et al., 2003), bi-polar disorder (Geller et al., 2004) and depression (Strauss et al., 2005; Hilt et al., 2007, but see also Strauss et al., 2004) have found an over-representation of Val allele homozygotes in these clinical samples. An initial study indicates this trend may extend to rumination. Hilt et al. (2007) found that among adolescent girls, those homozygous for the Val allele reported the highest rumination levels (Hilt et al., 2007). A primary goal of the current study therefore, was to test if this association between Val66Met genotype and rumination in adolescents would replicate in an independent sample of youth ranging from 8 to 14.

Given that rates of depression are comparatively low in childhood, but dramatically increase across adolescence (Hankin et al., 1998; Avenevoli et al., 2008), a potentially critical developmental consideration is whether genetic effects differ between children and adolescents. A number of studies have found that heritability of depression increases with age across childhood into adolescence (Schmitz et al., 1995; Murray and Sines, 1996; Scourfield et al., 2003). This trend is consistent with a genetic explanation for the increase in rates of depression during adolescence (cf. Birmaher et al., 1996; Lemery and Doelger, 2005) when rates of genetically linked behaviors may be inversely related to parental constraints and environments and behaviors are increasingly selected by maturing adolescents (Dick et al., 2007, 2009). The age-range in the current study, spanning across late childhood into early adolescence, enabled us to additionally test whether genetic associations differed between pre-pubescent children vs. young adolescents. In line with prior research, we hypothesized that genetic prediction of depression risk (the association between *BDNF* and rumination) would be significantly stronger among adolescents. Lastly, with a recent meta-analysis suggesting sex differences in the link between *BDNF* and depression risk (Verhagen et al., 2010) we also tested whether sex would moderate the association.

A secondary goal of the current study was to explore whether we could identify genetic markers of a related construct to rumination, an interpersonal intermediate phenotype of depression. Co-rumination is defined as the tendency to engage in extensive, negatively focused discussion with peers regarding reactions to and consequences of ongoing problems, to the exclusion of other activities (Rose, 2002). There is mounting evidence supporting the hypothesis that co-rumination is a maladaptive interpersonal tendency predictive of depression risk in youth and adolescence (Hankin et al., 2010; Stone et al., 2010, 2011).

Co-rumination is comprised of two distinct, non-overlapping constructs: the interpersonal tendency to engage in self-disclosure in response to ongoing problems, and the hypothesized consequence, the cognitive tendency to ruminate. Results suggest that co-rumination is indeed associated with both heightened levels of self-disclosure and rumination, but also reflects a distinct, separate construct (Rose, 2002). In deriving a theoretical rationale for a genetic predictor, we first determined co-rumination should share similar genetic markers with related constructs. Thus, in line with our primary hypothesis, we included the Val66Met polymorphism, anticipating that *BDNF* would contribute to the prediction of the cognitive-ruminative component of co-rumination. Secondly, we concluded that any single candidate polymorphism is unlikely to contribute enough predictive utility to capture the dual composition of the construct, leading us to a cumulative genetic score (CGS) approach composed of multiple polymorphisms.

In developing a CGS involving Val66Met, we considered the extensive research supporting that a functional polymorphism (5-HTTLPR) in the promoter region of the serotonin transporter gene predicts heightened reactivity to negative events, increasing depression risk (Caspi et al., 2003; for reviews see Caspi et al., 2010; Uher and McGuffin, 2010; Karg et al., 2011, but see also Munafò et al., 2009; Risch et al., 2009 for controversy over the gene × environment studies). Of particular import, research supports a biologic epistatic role of *BDNF* with 5-HTTLPR for the prediction of depression risk (Pezawas et al., 2008). Although studies have failed to support an association between 5-HTTLPR and rumination, either directly or in interaction with *BDNF*, the Val66Met and 5-HTTLPR combination has demonstrated utility in predicting cognitive reactivity to life events and mood inductions (Wells et al., 2010; Clasen et al., 2011). Therefore, the current study tests whether a CGS composed of both Val66Met and 5-HTTLPR would extend to the prediction of co-rumination (conceptualized as an interpersonal reaction to stressors). We did not anticipate a genetic association between the CGS and rumination based on prior research.

The CGS in the current study was composed of the Val66Met and 5-HTTLPR, by summing the number of “risk alleles” present. Thus, for *BDNF* we coded the number of Val present. Regarding 5-HTTLPR, a triallelic differentiation was made, such that short (S) alleles as well as a single nucleotide polymorphism (rs25531) in the long allele resulting in a G to A substitution (L_G_ vs. L_A_) was noted. Since prior research indicates that both S and L_G_ alleles exhibit reduced expression (Hu et al., 2005), we coded the number of either S or L_G_ alleles present as higher risk which aligns with prior work (e.g., Zalsman et al., 2006). We hypothesized that youth with higher genetic risk scores would report higher levels of co-rumination, and also explored whether participant age (children vs. adolescents) and sex would moderate this association.

Finally, given concerns over the replicability of candidate genetic polymorphisms with measures of psychopathology, and publication bias for positive associations, we apply a number of recently suggested standards toward the goal of introducing valid, replicable results by minimizing the likelihood of false-positive findings (Johnston et al., 2013). Specifically, we selected an established psychiatric phenotype, provide biological and psychological rational for selected polymorphisms, consider the impact of population heterogeneity, and address the need for novel genetic associations (co-rumination and CGS) to replicate. Hypotheses were first tested in a primary sample of 228 youth (Study I). In order to increase our confidence in these findings, we sought to gather supporting evidence in a small pilot study, which had identical recruitment methods (Study II). It is noteworthy that we previously published a non-significant association between BDNF and rumination in this sample (Gibb et al., 2012). However, we previously had not considered the potential moderating effect of age. Given mounting evidence suggesting differential genetic effects across the developmental stage from childhood into adolescence, we concluded this was an ideal sample to conduct replication tests.

## Materials and methods

### Eight- to 14-year-old children

In Study I, 255 mothers and their 8–14 year old children were recruited from the community. To qualify for inclusion, mothers either had to meet criteria for a major depressive episode (MDD) during the child's lifetime (*n* = 129) according to the *Diagnostic and Statistical Manual of Mental Disorders-Fourth Edition* (*DSM-IV*; American Psychiatric Association, 1994), or report no lifetime diagnosis of any *DSM-IV* mood disorder (*n* = 126). Exclusion criteria for both groups included symptoms of schizophrenia, organic mental disorder, alcohol or substance abuse within the last 6 months, or history of bipolar disorder. Youth's participation was limited such that no more than one child per mother could participate and all children were between the ages of 8–14 years. Study I analyses were based on 228 youth with complete assessment and genetic data. The average age of mothers in our sample was 40.36 years (*SD* = 6.83, Range = 24–55) and 86% were Caucasian. The median family income was $40,000–45,000 and, in terms of education level, 41% of the mothers had graduated from college. The average age of youth in Study I was 11.02 years (*SD* = 1.90), 53% were girls. Mothers' reported youths' race, with 82% of youth Caucasian, 6% African American, 1% Asian/Pacific Islander, 1% Hispanic, and 10% Bi-racial or Other.

Replication analyses were conducted in Study II, which had an identical recruitment strategy and criteria for inclusion, except youth ranged from 8 to 12 years of age. In Study II, 100 Mothers were recruited based on history of MDD during their child's life (*n* = 52) vs. no history of any mood disorder (*n* = 48). Additional information on recruitment and sample characteristics are available (Gibb et al., 2012). Study II analyses in the current manuscript were based on 87 youth with complete genetic data. Co-rumination data was collected during a follow-up conducted ~21 months later in which all participants were invited to complete. Of the 87 with complete genetic data from baseline, 65 participated. In Study II, the average age was 9.95 years (*SD* = 1.26), 60% were girls. Regarding race, mothers' reported that 81% of youth were Caucasian 6% African American, 2% Asian/Pacific Islander, 1% Hispanic, and 10% Bi-racial. In both studies, maternal history of MDD was not significantly related to children's age, sex, or race (Caucasian vs. non-Caucasian).

### Procedure

Potential participants for Study I were recruited from the community via newspaper ads. Mothers responding to the recruitment advertisements were initially screened over the phone to determine potential eligibility before being invited to participate. Upon arrival at the laboratory, mothers were asked to provide informed consent and youth were asked to provide assent to be in the study. Once consent and assent were obtained, mothers and youth completed diagnostic interviews and questionnaires in separate rooms. The recruitment and procedure of Study II mirrors the above outlined format with one exception. All data presented from Study II was collected at the initial assessment except for co-rumination, which was collected during a follow-up assessment that all families were invited to participate in.

### Measures

The SCID-I is a widely used diagnostic interviews with good psychometric properties (Zanarini and Frankenburg, 2001; First et al., 2002; Lobbestael et al., 2011) that was used to assess participants' history of Axis I diagnoses in Study I. The Schedule for Affective Disorders and Schizophrenia-Lifetime Version (SADS-L; Endicott and Spitzer, 1978) was administered to determine lifetime histories of Diagnostic and Statistical Manual of Mental Disorders (4th ed. [DSM–IV]; American Psychiatric Association, 1994) Axis I disorders in mothers. The SADS-L is a widely used diagnostic interview with substantial evidence to support its reliability and validity (Endicott and Spitzer, 1978). To assess inter-rater reliability, a subset of interviews (21 SCID interviews in Study I; 20 SADS-L interviews in Study II) were coded by a second interviewer, and kappa coefficients for diagnoses of MDD were excellent in both Study I (κ = 1.00) and Study II (κ = 1.00).

Children's depressive symptoms were assessed using the Children's Depression Inventory (CDI; Kovacs, 1981). The CDI is a 27-item self-report questionnaire that measures the presence of depressive symptoms in youth from 7 to 17 years of age. Each item presents three statements (coded 0–2) reflecting increasing symptom severity. Youth indicate which sentence best describes how they have felt over the past 2 weeks. The CDI is the most widely used depression rating scale in children and has demonstrated strong internal reliability, retest reliability (e.g., Tram and Cole, 2006) and convergent validity with other measures of depressive symptoms (for a review, see Klein et al., 2005). The CDI exhibited good internal consistency in both Study I (α = 0.86) and Study II (α = 0.85).

#### Risk factors

In Study I, brooding rumination in youth was assessed with the Children's Response Styles Scale (CRSS: Ziegert and Kistner, 2002). The CRSS is a 20-item questionnaire measuring youths' response styles (rumination vs. distraction) when confronted with feelings of depression. The brooding subscale is composed of 5 items (e.g., “When I feel sad, I think back to other times I felt this way”). Participants respond to items using an 11-point Likert scale ranging from *never* to *always*. Brooding rumination scores are calculated by summing all 5 items. The rumination subscale has demonstrated reliability and validity in youth and adolescents (Ziegert and Kistner, 2002). The brooding rumination subscale exhibited adequate internal consistency (α = 0.67).

In Study II, the brooding subscale from the Ruminative Response Scale (RRS-B; Treynor et al., 2003) was used to assess levels of brooding rumination in youth. The RRS is a self-report questionnaire that asks participants to rate the frequency with which they think or do certain things when they feel sad, down, or depressed (e.g., “Go someplace alone to think about your feelings”). The statements are rated on a 4-point Likert-type scale from *almost always* to *almost never*. The brooding subscale is composed of 5 items and has exhibited good reliability and validity in youth (e.g., Kuyken et al., 2006; Papadakis et al., 2006; Nolen-Hoeksema et al., 2007) samples. The internal consistency of the RRS-Brooding subscale was α = 0.62 for children.

In both studies, the Co-Rumination Questionnaire (CRQ; Rose, 2002) was used to assess youths' tendency to co-ruminate with peers. The CRQ is a 27 item self-report survey that assesses the extent to which participants co-ruminate with their closest same-sex friend. Items were designed to assess an extreme form of self-disclosure that exclusively focuses on negative events or problems. For example, “If one of us has a problem, we will spend our time together talking about it, no matter what else we could do instead.” Youth respond to items using a 5-point Likert-type scale ranging from “Not at all true” (1) to “Really true” (5). Total co-rumination scores are calculated by averaging participants' ratings across the 27 items. The CRQ has demonstrated moderate retest reliability (6-months, *r* = 0.54 Rose et al., 2007), and excellent internal consistency (α*s* = 0.96–0.97; Rose, 2002; Rose et al., 2007; Byrd-Craven et al., 2008). Internal consistency was excellent in Study I (α = 0.97) and Study II (α = 0.97).

#### Genetic data

Genomic DNA was isolated from buccal cells and saliva using a modification of published methods (Lench, 1988; Meulenbelt et al., 1995; Spitz et al., 1996; Freeman et al., 1997). The cheeks and gums are rubbed for 20 s with three sterile, cotton-tipped wooden swabs. The swabs are placed in a 50-ml capped polypropylene tube containing lysis buffer (500 μ l of 1 MTris–HCl; pH 8.0; 500 μl of 10% sodium docecyl sulfate; and 100 μ l of 5 M sodium chloride). The participants then rinse out the mouth vigorously with 10 ml of distilled water for 20 s and this was added to the 50-ml tube. Samples were stored at 4°C until the DNA was extracted. The Val66Met polymorphism (rs6265) was genotyped using Taqman assay C___11592758_10 (Applied Biosystems) using an ABI 7300 Real time PCR system. Study I's 228 participants were found to have the following *BDNF* genotypes:, Met/Met (10), Val/Met (71), and Val/Val (147). In Study II the *BDNF* data for the 87 subjects were as follows: Met/Met (4), Val/Met (25), and Val/Val (58).

The assay for 5-HTTLPR is a modification of that used by Lesch et al. (1996). The primer sequences are: forward, 5′-GGCGTTGCCGCTCTGAATGC-3′ (fluorescently labeled), and reverse, 5′-GAGGGACTGAGCTGGACAACCAC-3′ with yield products of 484 or 528 bp. Allele sizes are scored by two investigators independently and inconsistencies were reviewed and rerun when necessary. To distinguish between the S, LA, and LG fragments, the PCR fragment was digested with MspI by methods described in Wigg et al. (2006). Consistent with previous research (e.g., Zalsman et al., 2006), three groups of participants were formed based on their genotyping: (1) SS: children with two copies of the lower expressing 5-HTTLPR alleles (SS, SLG, or LGLG; *n* = 52), (2) SL: children with one copy of a lower expressing 5-HTTLPR allele (SLA or LGLA; *n* = 144), and (3) LL: children homozygous for the higher expressing LA allele (LALA; *n* = 52). The 228 participants in Study I were coded as follows (*n* = 49, 134, 45). The 87 participants in Study II exhibited similar trends in 5-HTTLPR data (*n* = 22, 45, 20), respectively.

## Results

Given the presence of missing data in the smaller replication sample (Study II: ≤ 25% missing across measures), we first examined whether data were missing at random, thereby justifying the use of data imputation methods for estimating missing values via sample averages (Schafer and Graham, 2002). Little's missing completely at random (MCAR) test, for which the null hypothesis is that the data are MCAR (Little and Rubin, 1987) was non-significant, χ^2^ (99) = 93.37, *p* = 0.64, supporting the imputation of missing values for Study II via expectation-maximization. Descriptive statistics of genotypes and study variables in both data-sets are presented in Table 1. Ruminative brooding did not differ according to youths' gender (*sr* = 0.06, *p* = 0.39; *sr* = 0.15, *p* = 0.16) or age (*sr* = −0.05, *p* = 0.49; *sr* = −0.03, *p* = 0.82) in either Study I or Study II, respectively. Regarding co-rumination, girls reported higher levels in Study I (*sr* = 0.24, *p* <0.001), and the effect trended toward significance in Study II (*sr* = 0.20, *p* = 0.07). Co-rumination levels did not differ according to age in Study I (*sr* = −0.07, *p* = 0.31), but in Study II co-rumination levels were significantly increased with age (*sr* = 0.22, *p* = 0.04). Age and gender did not significantly interact to predict differences in either outcome variable (lowest *p* = 0.37).

**Table 1:** **Descriptive statistics of participants from primary study (I) and replication study (II)**.

	**Study I *N* = 228**	**Study II *N* = 87**
Youth age	11.02 (1.90)	9.95 (1.26)
Youth race (% Caucasian)	82%	81%
Youth sex (% female)	53%	60%
Brooding rumination: CRSS	27.22 (9.78)	–
Brooding rumination: RRS	–	9.63 (2.80)
Co-rumination	2.71 (0.97)	2.23 (0.84)
Depressive symptoms	6.26 (6.04)	6.51 (6.36)
*BDNF* (%Met alleles: 0, 1, 2)	65, 31, 4%	67, 29, 4%
*5-HTTLPR* (% S or LG alleles: 0, 1, 2)	21, 59, 20%	23, 52, 25%

Hypotheses were tested using multiple linear regression analyses using Statistical Package for the Social Sciences (SPSS, IBM Corp, 2011). To account for recruitment strategy, all analyses were run with Mother's lifetime history of major depressive disorder as a dichotomous covariate. Thus, initial tests of genetic associations included genetic predictor and maternal depression history. Regressions testing age or gender moderation were run separately, entering the potential moderator as a third main effect, and the interaction term on the second level (Gene × Age). Tests of Gene × Age × Gender moderation included all main effects and lower order interactions entered on the first and second levels, respectively. Permutation testing (using 100,000 replications) was also used to compute the sampling distribution under the null hypothesis that the individual genetic variants or CGS has no effect on brooding rumination and co-rumination.

### Aim 1: BDNF and brooding rumination

Given the low rate of Met homozygotes, for single polymorphism analyses *BDNF* groups were dichotomized as Met carriers vs. Val homozygotes. Analyses predicting brooding rumination as the outcome variable are presented in Table 2. Focusing first on the primary sample (Study I), the association between *BDNF* genotype and rumination was not significant in the overall sample. To determine if Hilt et al. (2007) finding in adolescents would replicate, we tested age moderation by dichotomizing the sample between children (ages 8–10, *n* = 101) and adolescents (ages 11–14, *n* = 127). The *BDNF* × Age interaction was significant, *t*(223) = −2.27, *p* = 0.02, *sr* = −0.15, empirical *p*-value = 2.52E-2, with Val66Val genotype associated with higher levels of brooding rumination in adolescents *t*_(124)_ = −2.90, *p* < 0.01, *sr* = −0.25, empirical *p*-value = 4.30E-3. In contrast an association was not supported in children. Tests of sex (*BDNF* × Sex) and age by sex moderation (*BDNF* × Age × Sex) were not significant.

**Table 2:** **Genetic associations with brooding rumination: multiple linear regressions test whether *BDNF* (Val/Val genotype vs. Met allele carriers) directly in moderation with age or gender, predicts youths' tendency to ruminate**.

**Predictors**	**Study I (*N* = 228)**			**Study II (*N* = 87)**		
	***df***	***t***	***sr*_effectsize_**	***df***	***t***	***sr*_effectsize_**
*BDNF*	225	−1.51	−0.10	84	−0.09	−0.01
*BDNF* × Age	223	−2.27	−0.15^*^	82	−1.42	−0.15
Children (*n* = 101, 58)	98	0.51	0.05	55	0.83	0.11
Adolescents (*n* = 127, 29)	124	−2.90	−0.25^**^	26	−1.03	−0.20
*BDNF* × Sex	223	−0.19	−0.01			
*BDNF* × Age × Sex	219	0.08	0.01			
**SPECIFICITY TESTS**						
*5-HTTLPR*	225	0.43	0.03			
*5-HTTLPR* × Age	223	−0.46	−0.03			
*5-HTTLPR* × Sex	223	−0.60	−0.04			
*5-HTTLPR* × Age × Sex	219	−0.06	0.00			
CGS	225	1.34	0.09			
CGS × Age	223	1.07	0.07			
CGS × Sex		−0.25	−0.02			
CGS × Age × Sex	219	0.13	0.01			
**SENSITIVITY TEST**						
*BDNF* in adolescents	122	−2.65	−0.21^**^			

** p = 0.01,

* p = 0.05.

Next, replication tests were conducted in Study II. The *BDNF* × Age interaction did not reach statistical significance *t*(82) = −1.42, *p* = 0.16, *sr* = 0.15. Considering the small sample size may have been under-powered to detect the modest association found, we proceeded to test the association between *BDNF* and rumination separately in children and adolescents. Among adolescents, an association similar in effect size to Study I was exhibited, *t*(26) = −1.03, *p* = 0.31, *sr* = −0.20, however, it was not statistically significant in this sample.

Finally, tests of specificity and sensitivity were limited to Study I, given that the association did not reach statistical significance in Study II, which precluded comparisons. First, regarding specificity, there was no evidence that 5-HTTLPR was associated to rumination in the overall sample, or in interaction with age. Similarly, the CGS did not contribute to the prediction of youths' tendency to ruminate. Thus, results supported specificity of adolescent rumination to *BDNF* in this sample. Secondly, sensitivity or robustness of the association was tested by re-running the regression, covarying for variables commonly correlated with rumination: depressive symptoms (*r* = 0.22, *p* < 0.001) and co-rumination (*r* = 0.25, *p* < 0.001). The association between *BDNF* and rumination was maintained, *t*(122) = −2.65, *p* < 0.01, *sr* = −0.21, empirical *p*-value = 1.61E-2 suggesting the finding was not due to shared variance with these variables.

### Aim 2: CGS and co-rumination

Since results supported the initial hypothesis that Val66Val genotype would be associated with rumination in adolescents, we proceeded to the subsequent analyses testing for genetic association with co-rumination. Analyses predicting co-rumination as the outcome variable are presented in Table 3. First, analyses in Study I are presented testing genetic associations between *BDNF* and 5-HTTLPR with co-rumination individually. None of these tests were significant. Next we tested whether a CGS composed of *BDNF* and 5-HTTLPR would exhibit utility in predicting co-rumination. The CGS was created by summing the number of Val alleles present (0, 1, 2) from *BDNF* genotype with the number of lower-expressing S or L_G_ alleles (0, 1, 2) present from 5-HTTLPR. This yielded a genetic predictor of susceptibility ranging from 0 to 4. In Study I, the CGS was not significantly associated with co-rumination levels in the overall sample, nor in moderation by age (CGS × Age) or sex (CGS × Sex). However, the three-way interaction (CGS × Age × Sex) was significant, *t*(219) = 2.19, *p* = 0.03, *sr* = 0.14, empirical *p*-value = 3.55E-2. Exploring the break-down of this interaction, a significant association was found among adolescent girls, *t*(59) = 2.34, *p* = 0.02, *sr* = 0.28, empirical *p*-value = 2.66E-2, such that girls with higher CGS scores exhibited higher tendencies to co-ruminate.

**Table 3:** **Genetic associations with co-rumination: multiple linear regressions test whether the CGS directly or in moderation with age or gender, predicts youths' tendency to co-ruminate**.

**Preliminary tests**	**Study I (*N* = 87)**			**Study II (*N* = 228)**		
	***df***	***T***	***sr*_effectsize_**	***df***	***t***	***sr*_effectsize_**
*BDNF*	225	0.72	0.05			
*BDNF* × Age	223	−1.05	0.07			
*BDNF* × Sex	223	−1.88	−0.12			
*BDNF* × Age × Sex	219	−0.78	−0.05			
*5-HTTLPR*	225	1.89	0.12			
*5-HTTLPR* × Age	223	−1.69	−0.11			
*5-HTTLPR* × Sex	223	0.28	0.02			
*5-HTTLPR* × Age × Sex	219	1.25	0.08			
**CUMULATIVE GENETIC SCORE TESTS**						
CGS	225	0.86	0.06	84	1.26	0.14
CGS × Age	223	−0.40	−0.03	82	−1.08	−0.11
CGS × Sex	223	1.52	0.10	82	0.92	0.10
CGS × Age × Sex	219	2.19	0.14^*^	78	−0.25	−0.03
Children M (*n* = 43, 9)	40	1.00	0.16	6	0.12	0.05
Children F (*n* = 58, 13)	55	0.40	0.05	10	1.73	0.47
Adolescents M (*n* = 65, 26)	62	−1.66	−0.20	23	0.21	0.04
Adolescents F (*n* = 62, 39)	59	2.34	0.28^*^	36	2.04	0.31^*^
**SENSITIVITY TEST**						
CGS in Adolescents F	57	2.22	0.25^*^	34	1.89	0.29

* p = 0.05.

Replication tests of this novel finding were run in Study II. The interaction (CGS × Age × Sex) was not significant. Exploratory tests of the association among subgroups yielded a similar pattern, such that CGS was only a significant predictor of co-rumination among adolescent girls *t*(36) = 2.04, *p* < 0.05, *sr* = 0.31, with the empirical *p*-value = 5.56E-2 suggesting a significant trend.

To determine the sensitivity of the association between CGS and co-rumination in adolescent girls, we re-ran the regression co-varying for the effects of concurrent depressive symptoms and rumination. In Study I the association was maintained, *t*(57) = 2.22, *p* = 0.03, *sr* = 0.25, empirical *p*-value = 4.86E-2. In Study II, after covarying for baseline depressive symptoms and rumination the association trended toward significance, *t*(34) = 1.89, *p* = 0.08, *sr* = 0.29, empirical *p*-value = 7.53E-2. Finally, to test specificity of the CGS and co-rumination association we explored whether CGS would predict adolescent rumination in Study I. Results did not support an association (Table 2).

### Additional tests of reliability

Two potential sources of variance in both studies include the composition of participants, and recruitment strategy. Regarding ethnicity, the majority of participants in both studies were Caucasian. Although *BDNF* and 5-HTTLPR genotypic variation did not significantly differ according to race (Caucasian vs. not) in either sample, the results presented were re-run co-varying for child's race (Caucasian vs. not). None of the outcomes differed significantly. Secondly, given that maternal depression could account for important variance, analyses were also rerun without covarying for this effect. In Study II, the link between CGS and co-rumination in adolescent girls was no longer significant when maternal depression was not a covariate, *t*(37) = 1.20, *p* = 0.24, *sr* = 0.19, empirical *p*-value = 2.36E-1. We are hesitant to interpret this finding, given none of the analyses in Study I differed after testing both sources of heterogeneity.

## Discussion

The goal of the current study was to identify genetic markers of depression risk in youth by focusing on endophenotypes. Specifically, the study sought to replicate an initial genetic association between *BDNF* and rumination in adolescents, and extend this association to the prediction of co-rumination via a CGS approach. Results supported that the Val66Met SNP was associated with adolescents' brooding rumination, such that adolescents homozygous for the Val allele reported higher levels of brooding than carriers of the met allele, replicating Hilt et al. (2007) initial finding.

An exciting aspect of the current study design was the ability to explore the specificity and sensitivity of the *BDNF*-rumination link. Results indicated support for specificity in genetic prediction, such that analyses in Study I did not reveal an association between 5-HTTLPR and rumination. This finding aligns with prior work (e.g., Beevers et al., 2009), although there is some evidence 5-HTTLPR may moderate the impact of life stress on rumination (Canli et al., 2006; Clasen et al., 2011). Importantly, results also provide initial support that the genetic association to brooding rumination is not attributable to overlap with other behavioral phenotypes including depressive symptoms and co-rumination. Given that these tests were limited to Study I, they should be interpreted with caution pending replication in future research.

The current association found between Val66Val and rumination in adolescents, aligns with prior work supporting that youth and adolescents with Val/Val genotype are more likely to ruminate (Hilt et al., 2007) and develop depression, mood and anxiety disorders (Hall et al., 2003; Geller et al., 2004; Strauss et al., 2005; Hilt et al., 2007). In contrast, studies on adult depression have found that Met allele carriers are over-represented in clinical populations (Jiang et al., 2005; Hilt et al., 2007) or exhibit higher rumination or depressive symptoms in community samples (Beevers et al., 2009; Duncan et al., 2009), but see also Juhasz et al. (2011). The failure to distinguish between adult vs. childhood onset of depression has been proposed to account for the lack of association found between Val66Met and lifetime depression in meta-analyses (e.g., Gratacos et al., 2007; Chen et al., 2008). Although it has been argued that *BDNF* may have differential effects according to developmental stage, this continues to be a controversial issue, since other studies that have distinguished timing of onset, have also failed to establish an association in adult populations (Hong et al., 2003; Strauss et al., 2004).

It is noteworthy that the Val66Val and rumination correlation was only significant among young adolescents, in comparison the association was not supported among older children. We are hesitant to interpret the age moderation test, which implies a significant difference in genetic prediction between youth and adolescents. However, the findings align with prior research supporting differential genetic prediction, such that heritability of depression increases from childhood into adolescence (Schmitz et al., 1995; Murray and Sines, 1996; Scourfield et al., 2003). One explanation for this effect is that the increase in genetic contribution across this specific developmental span coincides with the timing in maturation of risk factors or endophenotypes. The advanced cognitive styles associated with depression (e.g., negative attributional style, dysfunctional attitudes) demonstrate increasing stability with age, reflecting variable, state-like characteristics in youth that stabilize to reflect more consistent traits across adolescence (e.g., Cole et al., 2008; Hankin, 2008). However, this explanation does not fit with research on rumination, which has been found to exhibit relatively low stability in adolescence (Hankin, 2008), and exhibits utility as a predictor of depression risk in both children and adolescents (Gibb et al., 2012). Another possibility is that genetic contribution increases during adolescence due to onset of puberty and associated changes. Specifically, it has been postulated that differential neurobiological development of the amygdala relative to the slower, progressive maturation of the prefrontal cortex has been found to account for adolescents' heightened mood liability, impulsiveness, and level of emotion regulation (Casey et al., 2008). Therefore, genetic influences on neural processes that would otherwise be modulated by a more established executive function might manifest in greater behavioral extremes in adolescence compared to childhood. Finally, it is also possible that the increase in heritability during adolescence may reflect a co-occurring decrease in environmental variance as adolescents select their surroundings (which are largely determined by parents in childhood). Future research is needed to confirm and replicate the timing of this differential effect, and explore these possibilities to determine the causal factors of this emerging trend.

A second aim of the current study was to extend genetic markers of depression risk to the prediction of co-rumination. Results indicate that a CGS comprising Val66Met and 5-HTTLPR predicts adolescent girls' tendency to co-ruminate, with the presence of greater risk alleles (Val and S or L_G_) associated with higher co-rumination levels. Further, results supported tests of sensitivity, such that the genetic association with co-rumination was not attributable to adolescents' tendency to ruminate, or concurrent depression. The utility of a combined Val66Met and 5-HTTLPR genetic predictor is based on research supporting biologic epistasis for predicting depression risk (Pezawas et al., 2008) and is consistent with more recent studies predicting cognitive vulnerability to depression in the context of environmental stress (Wells et al., 2010; Clasen et al., 2011).

In the primary study, this genetic association was moderated by both participant age and gender, with the CGS only a predictor of adolescent girls' tendency to co-ruminate. Gender differences in genetic associations may be interpreted as genes having different biological effects on each sex. Alternatively gender moderation could reflect differences in environmental shaping (gender socialization) of behaviors and thus may be viewed as an environmental moderator. A recent study supports the latter, with resilience found to have higher heritability among men than women, which was found to be partially attributable to gender differences in environmental experiences (Boardman et al., 2008). We interpret a similar effect in the current study. That is, although co-rumination is predictive of depressive risk in both genders (Hankin et al., 2010; Stone et al., 2010, 2011), girls are more interpersonally-oriented, and co-rumination is much more common tendency among girls' friendships than boys' (Rose, 2002; Hankin et al., 2007; Rose et al., 2007). Further, co-rumination also increases with age, and thus is most prevalent among adolescent girls (Hankin et al., 2010; Stone et al., 2010, 2011). However, this is a novel finding, and we are hesitant to conclude age and gender moderation effects pending successful replication.

A unique aspect of the study was the ability to test whether results were supported by analyses in an independent sample. This is particularly important given the publication bias for positive or novel genetic association findings, and higher likelihood of publishing novel results from small samples yielding random effects (e.g., Johnston et al., 2013). As previously reported, (Gibb et al., 2012) in Study II the link between *BDNF* and rumination was not significant in the overall sample, and when tested specifically among adolescents the result still did not reach statistical significance according to the *p* = 0.05 criterion. However, in line with advocates of prioritizing tests of effect-sizes rather than reliance on null hypothesis significance tests (Cohen, 1994), the consistency of the effect-sizes across both studies is compelling (Study I, *N* = 127, *r*_effectsize_ = 0.25 vs., *r*_effectsize_ = 0.20 in Study II, *N* = 28), especially in the context of the permutation analyses which suggest that the pattern of results observed in both studies are not likely to be driven by random effects. Importantly these results also mirror the initial finding (Hilt et al., 2007;*N* = 91, *r*_effectsize_ = 0.25). Focusing next on the replication test of the association between co-rumination and CGS, Study II did not replicate the age by gender moderation result found in Study I. However, when testing among subgroups, a significant association was found specific to adolescent girls. Therefore, although the genetic associations exhibited strikingly consistent effect-sizes among specific sub-samples, the small sample sizes increase the possibility of chance findings. Therefore, future work is needed to test whether age and gender moderation effects replicate in large, independent samples.

The current study benefits from the application of recent standards for candidate gene association studies (Johnston et al., 2013), with theory-driven selection of established psychiatric phenotypes, polymorphisms, and access to two samples with identical recruitment methods. However, several limitations should be noted. First, given that several moderators were explored, both studies would have benefited from larger sample sizes. Additionally, a limitation to Study II was that co-rumination was measured at a separate assessment from the other baseline measures. Although we argue that the consistency in genetic associations in Study II is compelling based on similarity of effect-size, it will be important to confirm that results replicate in independent samples adequately powered to capture the small effect-size seen in this genetic association. Further, given that genotype frequencies differ according to ethnicity, future research with larger, diverse samples is needed to determine if results generalize beyond Caucasian samples. Next, although the study successfully replicated the association between the Val66Met SNP and rumination in adolescents, a more thorough analysis may incorporate haplotypes to further clarify the link between *BDNF* and depression risk. Further, specificity tests of genetic associations could be extended by accounting for other candidate genes implicated in depression risk (e.g., *HOMER1*, *TPH*, *DRD4*, *COMT*). Although the CGS exhibited utility in the current sample, there were limitations to our approach including an assumption of equal weights between known genes, and reliance on an additive model that did not allow for modeling of epistasis or consideration of environmental moderators. Finally, there is always the possibility in any genetic association study of an unmeasured genetic or non-genetic third variable accounting for the associations reported (e.g., unaccounted for population stratification or linkage disequilibrium between measured variant and actual functional variant). Future studies, therefore, would benefit from the inclusion of genomic control.

In summary, the current study replicates Hilt et al. (2007) genetic association between *BDNF* and rumination in adolescents, and offers initial evidence suggesting an association between Val66Met and 5-HTTLPR and co-rumination in adolescent girls. While there is consistent evidence across the subpopulations in the test and replication samples, the small sample sizes of these studies stress the need for larger studies of this nature that can replicate these findings with even greater confidence and consider differential genetic effects according to gender and developmental state. Findings support the utility of candidate gene and aggregate risk approaches for predicting cognitive and interpersonal phenotypes of depression risk in adolescence.

### Conflict of interest statement

The authors declare that the research was conducted in the absence of any commercial or financial relationships that could be construed as a potential conflict of interest.
